# Risk Factors of Anaemia among Children under Five Years in the Hohoe Municipality, Ghana: A Case Control Study

**DOI:** 10.1155/2019/2139717

**Published:** 2019-06-25

**Authors:** Phyllis Atta Parbey, Elvis Tarkang, Emmanuel Manu, Hubert Amu, Martin Amogre Ayanore, Fortress Yayra Aku, Sorengmen Amos Ziema, Samuel Adolf Bosoka, Martin Adjuik, Margaret Kweku

**Affiliations:** ^1^School of Public Health, University of Health and Allied Sciences, Ho, Volta Region, Ghana; ^2^Department of Population and Behavioural Sciences, School of Public Health, University of Health and Allied Sciences, Ho, Volta Region, Ghana; ^3^Department of Family and Community Health, University of Health and Allied Sciences, Ho, Volta Region, Ghana; ^4^Department of Epidemiology and Biostatistics, University of Health and Allied Sciences, Ho, Volta Region, Ghana

## Abstract

**Background:**

Anaemia is one of the major causes of death among children under five years in Ghana. We examined the risk factors of anaemia among children under five years in the Hohoe Municipality, Ghana.

**Methods:**

This facility-based matched case control study recruited 210 children (70 cases and 140 controls) aged 6 to 59 months. Stratified and simple random sampling techniques were used to select mothers attending Child Welfare Clinic (CWC) for the screening of their children. Data were collected using a semistructured questionnaire. Finger prick blood was collected to estimate the haemoglobin (Hb) level and thick film was prepared to determine malaria parasitaemia. Axillary temperature was measured using an +electronic thermometer and anthropometric measurements were done using a weighing scale and inelastic tape measure. Continuous variables were presented as means and standard deviations and categorical variables as frequencies and proportions. Conditional logistic regression was used to determine the strength of association between the dependent and the independent variables. Statistical significance was considered at p value of <0.05.

**Results:**

The prevalence of anaemia was high (53.8%), while children whose mothers received iron supplementation during pregnancy were 7.64 times more likely to be anaemic compared with those who did not [AOR=7.64 (95% CI:1.41-41.20.93); p=0.018]. Children with poor dietary diversity were 9.15 times more likely to have anaemia [AOR=9.15 (95% CI: 3.13-26.82); p< 0.001]; and children whose mothers were farmers and traders were 83% [AOR = 0.17 (95% CI: 0.05-0.60); p=0.006] and 79% [AOR=0.21 (95% CI: 0.06-0.74); p=0.014], respectively, less likely to have anaemia.

**Conclusion:**

The biologic, intermediate, and underlying factors that were significantly associated with anaemia comprised maternal iron supplementation, poor dietary diversity, farmers, and traders. Given that iron supplementation during pregnancy did not protect children against anaemia, we recommend the child's nutritional dietary diversity is encouraged.

## 1. Introduction

Anaemia is one of the most serious and common nutritional deficiency disorders of public health concern in developing countries [1]. Approximately 50% of anaemia cases are due to iron deficiency, though the proportion varies among population groups and in different areas, according to the prevailing local conditions [2]. Anaemia is defined as a decrease in the concentration of circulating red blood cells concentration and a concomitant impaired capacity to transport oxygen [2, 3]. Anaemia diagnosis is classified as mild (Hb=10.0-10.9 g/dl), moderate (Hb=7.0-9.9 g/dl), severe (Hb<7.0g/dl), and normal (Hb≥11.0 g/dl) Hb level concentration for children aged 6 to 59 months [2].

Inspite of anaemia being preventable, it has caused a lot of morbidity and mortality in children under five years [4]. Anaemia has adverse effects on children, especially in the first two years of life, such as behavioural delay, reduced cognitive development (impaired learning and decreased school achievement), low immunity and growth weight, fatigue, difficulty with concentration, lethargy, increased mortality, and susceptibility to infection [2, 5, 6]. Common signs and symptoms of anaemia in children are dizziness, fatigue and body tension, general body weakness, loss of appetite, low body weight, paleness of the skin, eyes and palms, and under severe conditions, unconsciousness, and finally death [7].

Globally, it is estimated that 273 million (approximately 42.6%) of children under five years are anaemic, whilst 60.2% of children under five years in the African region are anaemic [2]. In Kenya, a population-based cross-sectional study revealed that low iron diet intake and malaria were the main causes of anaemia [5]. Parasitic infections (hook worm and tapeworm), acute or chronic inflammations, inherited or acquired disorders that affect Hb synthesis, red blood cell production, or red blood cell survival and nutritional deficiencies were other factors that were identified to be associated with anaemia among children under five years [8]. In a similar but unrelated study, it was established that unemployment among caretakers, malaria parasitaemia, and presence of sickle Hb were the risk factors associated with severe anaemia among children under five years in Tanzania [9]. Meanwhile, male sex, 9–11 months of age, poor dietary diversity, stunting, diarrhoea, early initiation of complementary food, and lowest wealth quintile were the risk factors associated with anaemia among children under five years in Ethiopia [10].

In Ghana, the prevalence of anaemia (percentage of children with Hb concentration <110 g/L) among children aged 6 to 59 months was 76% in the year 2011 and was reported to be of severe public health significance [2]. Although the prevalence of anaemia decreased, it only decreased with increase in age [7] meaning that children were still in danger of being anaemic. The prevalence for anaemia among children aged 6 to 59 months in the year 2014, according to Ghana Demographic and Health Survey [GDHS] (2014) report, was 66%, with the Volta Region's rate superseding the national prevalence rate (69.9%). As such, the Ministry of Health (MoH), Ghana, in partnership with the Ghana Health Service (GHS), and other private organizations have developed policies and interventions that are multisectoral and integrated approach to help prevent anaemia. These policies and programmes designed for the prevention of anaemia are mostly for children, pregnant women and women in reproductive age [11]. The targeted interventions for anaemia are malaria control which includes distribution and use of Long Lasting Insecticide treated Nets (LLINs), Indoor Residual Spraying (IRS), Intermittent Preventive Treatment of Pregnant Women (IPTp), and diagnosis, and iron folic acid supplementation to pregnant women and treatment of malaria with Artemisinine-based Combination Therapy (ACT).

As a result of the high prevalence rate of anaemia in children in the Volta Region, studies have been conducted among children aged less than five years in the Hohoe Municipality on the subject [12, 13]. The previous studies used cross-sectional approach to purposively sample four rural communities in the municipality to establish associated factors of anaemia among children under five years of age in the Municipality. However, our study sought to use a case-control approach sampling health facilities from all seven submunicipalities within the municipality to give a true representation of all communities on the causal-effect relationship between anaemia and its risk factors among the same study group. It also determines the Hb concentration level among children aged 6 to 59 months, the association between anaemia and underlying factors of anaemia, the association between anaemia and intermediate factors, and the association between anaemia and biological risk factors of anaemia.

### 1.1. Conceptual Framework

**Figure 1:**
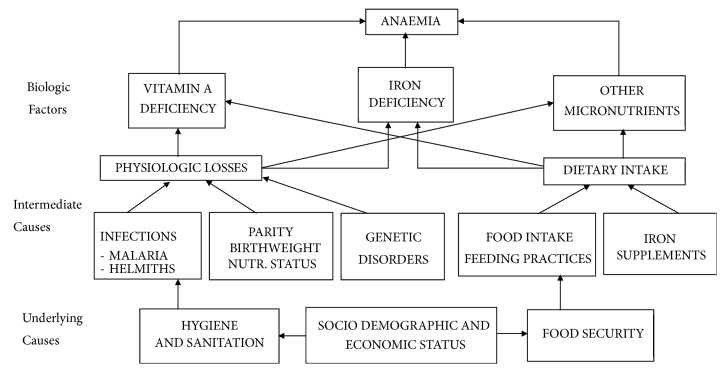
Conceptual framework for causes of childhood anaemia adapted from [14].

The conceptual framework adopted in this study explains the possible causes of anaemia and the interconnectedness of these factors in predisposing a child or an individual to the diseases (Figure 1). Household food security, access to healthcare and public policies, coupled with hygiene and sanitation issues, serves as underlying causes of anaemia among children. Infections from organisms such as helminths and malaria parasitaemia, parity, and birth weight of a child and genetic disorders cause physiological losses to the child, aggravated by dietary intake, resulting from feeding habits and food supplementation. These factors serve as the intermediate cause of anaemia among children under five years of age. The presence of these physiological factors activates the child's biological factors such as vitamin A deficiency, iron deficiency, and the deficiency of other essential micronutrients to cause anaemia in a child.

## 2. Materials and Methods

### 2.1. Setting

This study was carried out in Hohoe Municipality, which is one of the twenty-five administrative districts of the Volta Region of Ghana. It is bounded by Jasikan District to the North, Northwest by Biakoye District, South by Afadjato South District, West and South West by Kpando Municipality, and East by the Republic of Togo. The population of Hohoe Municipality, according to the 2010 Population and Housing Census, is 167,016 representing 7.9% of the total population of the Volta Region. It comprises 52.1% females and 47.9% males with a growth rate of 2.5%. The population of children below five years is 21,913 which is 12.4% of the population. The population of households in the Municipality is 164,326 and children represent 37.4 % [15].

There are two rainy seasons: major and minor. The major season starts from April and ends in August while the minor lasts from September to November. Farming (55%), trading (25%), and livestock rearing (15%) are the major occupations. The main cash crops are cocoa, maize, cassava, rice, yam, and various vegetables notably amongst them being “*nkontomire*”, tomatoes, pepper, and okro [15].

In terms of health services, the municipality has been divided into seven Health Sub-Municipalities, namely: Akpafu/Santrokofi, Alavanyo, Agumatsa, Lolobi, Gbi-Rural, Hohoe-Sub, and Likpe. Hohoe Municipality has a total of twenty-one health facilities including the Municipal Hospital (1), Health centres (14), and Community-based Health Planning and Services (CHPS) compounds (6). There are 57 Expanded Programme on Immunisation (EPI) outreach clinics which operate monthly [16].

### 2.2. Population and Sampling

The study population were children aged 6 to 59 months and their biological parents who resided in Hohoe Municipality, Volta Region, Ghana. The mothers of the children consented in order to allow their children to participate in this study. Stratified and simple random sampling techniques were used to recruit respondents for the study. The seven submunicipalities in the Hohoe municipality were stratified into urban and rural communities and one facility per stratum (urban and rural) was randomly selected by following simple random sampling procedures. In all, fifteen mothers, with children aged 6-59 months attending CWC, were randomly selected through balloting, per facility, after the purpose of the study has been explained to them. Five of the participants per facility were cases and appropriate controls were selected. The same procedure was followed in all the selected CWCs to achieve the required sample size. After screening for Hb concentration, the children were classified as cases (Hb<11g/dl) or controls (Hb≥11.0g/dl) using the WHO cut-off for Hb concentration [2]. A case was matched to two controls based on similar characteristics such as age ± 5 months, sex, and also resident in the same community.

The mothers were required to give the needed information for the purpose of the study. Children aged 6 to 59 months with Hb level <11.0 g/dl were considered as cases (children with anaemia). The parents of these children were residents in Hohoe Municipality for at least six months and also consented to participate in the study. Children aged 6 to 59 months with normal Hb level using the WHO Hb cut off (≥11.0 g/dl) were considered as controls in this study [2]. Also, parents of these children were residents in Hohoe Municipality for at least six months and consented to participate in the study.

Children who qualified to partake in the study but were seriously ill and required medical care were excluded. Moreover, eligible children who did not report at CWC with their mothers were excluded from this study. This is because the responses to some questions such as exclusive breastfeeding, intake of Sulfadoxine-Pyrimethamine (SP) and iron folate/folic acid during pregnancy by mother and child feeding practices would be appropriate if obtained from the mother. Children whose mothers did not consent to participate in the study were also excluded.

### 2.3. Design and Procedures

This was a facility-based matched case-control study conducted in March 2017 to determine the associated risk factors of anaemia among children under five years in Hohoe Municipality. The sample size for the study was calculated using the formula:

(1) $$\begin{array}{c} n=\left(\;\frac{r+1}{r}\;\right)\frac{\left(\overset{-}{p}\right)\left(1-\overset{-}{p}\right){\left(Z_{\beta }+Z_{\alpha /2}\right)}^{2}}{{\left(p_{1}-p_{2}\right)}^{2}} \end{array}$$

[17], where n is sample size for case group, 80% power Z_*β*=_0.84, and r=ratio of control to cases, 1 for equal number of case and control.

$\overset{-}{p}$=average proportion exposed=proportion of exposed cases + proportion of controls exposed/2.

*Z*_*β*_=standard normal variate for power= for 80% power it is 0.84 and for 90% value is 1.28.

*Z*_*α*/2_ =standard normal variate for level of significance Z_*α*_=1.96.

*P*_1_ − *P*_2_ = Effect size or different in proportion expected based on previous studies, *P*_1_ is proportion in cases, and *P*_2_ is proportion in controls using 47.5% [13] as the prevalence of cases, 26.6% among controls, and an assumed odds ratio of 0.4. Hence, a total of 70 cases and 140 controls were obtained.

A pretested semistructured questionnaire using face-to-face interview was used to obtain data on the sociodemographic characteristics, dietary diversity among mothers with children aged 6 to 59 months; clinical data and anthropometric measurements were obtained through the appropriate procedures.

Haemoglobin concentration was estimated using URIT-12 Hb (URIT medical Electronic Co., LTD, China). Finger pricked blood sample (20*μ*l) was collected from all the study subjects for measurement of Hb concentration. Children who had anaemia requiring treatment (Hb<8.0g/dl) were referred to the Hohoe Municipal Hospital for management and treatment.

The finger pricked blood sample was also used to prepare blood film for the detection of malaria parasitaemia. The blood sample was placed on a clean, dust-free frosted slide for thick blood film. The appropriate laboratory procedures were used to stain the slides with 1% Giemsa stain for 25-30 minutes and slides were examined under oil immersion with a light microscope (ocular magnification X100) by two microscopists. A sample was considered negative only after 200 high power fields had been read without any parasite seen. Parasites counts were converted to parasites per microliter, assuming a white blood cell count of 8,000 leukocytes per microliter of blood. All blood slides were stored in appropriately labelled slide boxes and kept at the laboratory.

Children found to have malaria parasitaemia were followed up for treatment with Artemether-Lumefantrine, one of the approved ACTs for treatment of uncomplicated malaria. Dosage needed for treatment for each child was based on the weight of the child as provided in the national treatment guideline.

Axillary temperature was measured using electronic thermometer (GH Zeal Ltd, London, UK) and children with body temperature ≥ 37.5°C and above were classified as having fever.

Nutritional assessment was done using nonstretchable tape to measure MUAC by estimating the MUAC to the nearest 0.1 centimetres (cm). The tape had three colours: red, yellow, and green. The red indicates severe acute malnutrition (11.0 cm), the yellow, moderate acute malnutrition (11.0-11.9 cm) and the green, and normal nutritional status (≥12.0 cm).

Regarding dietary diversity assessment (24 hours recall), dietary diversity was assessed using the WHO [18] recommended seven food groups used in defining children's minimum dietary diversity indicator as follows: (i) grains, roots, and tubers; (ii) legumes and nuts; (iii) dairy products; (iv) flesh foods (meats/fish/poultry); (v) eggs; (vi) vitamin A rich fruits and vegetables; and (vii) other fruits and vegetables will be used [18]. The WHO concept has been applied to children aged 6 to 23 months but in this study it was adapted to apply to children aged 6 to 59 months. The dietary diversity score (DDS) ranged from 0 to 7 with minimum of 0 if none of the food groups was consumed and 7 if all the food groups were consumed. In this study, adequate dietary diversity was defined as consumption of food from at least four different food groups (DDS ≥ 4).

### 2.4. Data Management and Analysis

Unique codes were assigned to participants and were used for the data entry. Data were entered into EpiData 3.1 software. The data was cleaned and exported to Stata version 14.1 for analysis. Simple frequencies and percentages were used for categorical variables. A univariate conditional logistic regression was used to determine the association of independent variables to the dependent variable. Using a manual stepwise forward selection of variables, significant variables were put in the multiple conditional logistic model, one at a time, in checking for significant association until a final model of significant variables was achieved. Statistical significance was considered based on p-value of <0.05.

### 2.5. Ethical Issues

Before commencement of the study, ethical approval was obtained from the University of Health and Allied Sciences (UHAS) Ethical Review Committee (ERC) with approval number UHAS-REC A.6 [7] 17/18. After obtaining ethical clearance from the UHAS ERC, permission and approval were sought from the Hohoe Municipal Health Directorate. The study was conducted in accordance with accepted principles on Ethics in Human Experimentation and International Conference on Harmonisation/Good Clinical Practice (ICH/GCP). Before inclusion into the study, written individual informed consent was obtained from the mothers of the children using the consent form approved by the ethics committee, after the scope and objectives of the study was thoroughly explained to participants.

## 3. Results

### 3.1. Demographic Characteristics of Children Aged 6 to 59 Months

Table 1 presents the sociodemographic characteristics of children included in the study. The mean ages for the cases and controls were 34.2±14.4 months and 34.7±14.2 months respectively. Eighty percent of the children were aged between 24 and 59 months. The cases cohort in this age category was 78.6% cases and 80.7% controls. Females were 52.9% of the cases. Similarly, females were 52.9% of the controls. With reference to birth rank of the children, those who were the first-borns (32.4%) was the majority, followed by the fourth-borns and above (25.2%), second-borns (23.3%), and the least were third-borns (19.0%). Seventy-nine percent of the children were delivered at a health facility, 75.7% of the cases, and 80.0% of the controls being born in health facilities. Majority of the children owned LLIN (95.7%) out of which 95.7% was so for cases and 95.7% for controls. Sleeping inside LLIN the night before the survey was 79.0%. A total of 80.0% of cases and 78.6% of controls did sleep inside LLIN the night before the survey. Only 5.2% of the children tested positive for malaria parasitaemia. Fever, defined as body temperature ≥37.5°C, was present in 6.7% of the children. Malaria parasitaemia was present in 5.7% of cases and 7.1% of controls. Overall, 7.1% were wasted of which 7.1% and 7.1% of cases and controls were so, respectively. Approximately 1.9% said they were sickle cell positive.

### 3.2. Socio Demographic Characteristics of Mothers

Majority (45.7%) of the mothers were aged between 20 and 29 years followed by 30-39 years (40.0%). Majority (91.9%) of the mothers were Christians. Most (76.7%) mothers were Ewes. Most (76.7%) of the mothers had attained primary/junior high school (JHS) educational level whilst 7.6% had no formal education.

The predominant occupation of the mothers was hairdressing/trading (53.3%). Majority (81.4%) of mothers were married and most (47.1%) of them had 1-2 children. Most (38.1%) mothers belonged to high socioeconomic status (SES) (Table 2).

### 3.3. Association between Biologic, Intermediate, and Underlying Factors and Anaemia (Conditional Logistic Regression)

A univariate analysis conducted between anaemia and each of the independent variables (use of LLIN, presence of malaria, sickle cell disease, maternal age, educational level, marital status, parity, monthly income, socio economic status, exclusive breastfeeding, difficulty swallowing, and source of drinking water) showed no significant association (p≥0.05). However, using a manual stepwise forward selection of variables in a multiple conditional logistic regression model it was found that occupation (farming and trading), dietary diversity score, and mother's iron supplementation were statistically significant as shown in Table 3.

Children whose mothers received iron supplementation during pregnancy were 7.6 times more likely to have anaemia as compared to those who did not and the difference was statistically significant [AOR=7.64 (95% CI: 1.41-41.20.93); p= 0.018]. Children with poor dietary diversity were 9.15 times more likely to have anaemia as compared to those with good dietary diversity and the difference was statistically significant [AOR=9.15 (95% CI: 3.13-26.82); p<0.001].

Children whose mothers were aged <20 years or between 30 and 39 years were 4.69 and 2.55 times, respectively, more likely to develop anaemia, as compared to those who were aged 40 years and above and the difference was not statistically significant [AOR=4.69 (95% CI:0.46-47.58); p=0.191] and [AOR=2.55 (95% CI:0.66-9.88); p=0.175], respectively. Children whose mothers were farmers and traders were 83% and 79% times less likely to have anaemia as compared to those who were unemployed and the differences were statistically significant [AOR=0.17 (95% CI:0.05-0.60); p=0.006] and [AOR=0.21 (95% CI:0.06-0.74); p=0.014], respectively.

## 4. Discussion

The prevalence of anaemia (Hb<11.0g/dl) among children aged 6 to 59 months was 53.8%. The finding on the prevalence is similar to the results of Semedo et al. [1] in Cape Verde, which revealed that 51.8% of children aged 6 to 59 months had anaemia. The prevalence of anaemia observed in this study was, however, lower compared to studies conducted in the Volta Region and in Uganda. Those studies found the prevalence to be 69.9% and 58.8%, respectively, among children aged 6 to 59 months [10, 19]. Although lower than the anaemia prevalence in the Region, the prevalence in this study was higher than that reported in a study conducted by Gari et al. [20] in Adami Tullu District, South-Central Ethiopia, where they found the prevalence of anaemia to be 36.8%. Similarly, a study undertaken in November 2016 by Kweku et al. [12] among children under five years residing in the rural communities in the same study area also showed anaemia prevalence to be 48.1%. The prevalence in the present study was higher than their prevalence. That study concentrated on rural dwellers only while this current study included both rural and urban dwellers. Hence, the differences in prevalence could be because of the environment, climatic and agricultural systems as compared to the study of Kweku et al. [12] which recorded lower prevalence probably due to the differences in seasons in which the studies were conducted. The study by Kweku et al. [12] was conducted at the end of the rainy season when food and vegetables were in abundance while this current study was carried out in March, during the dry season when food was scarce.

Considering the association between biologic factors and anaemia, the current study found that children whose mothers received iron supplementation during pregnancy were 7.64 times more likely to have anaemia compared with those who did not. The findings differ from what was found by Logan et al. [21]. In their study, the authors argued that mothers' adherence to oral iron administration was higher in the group of children without anaemia compared with those who were having anaemia. The provision of iron supplementation to women in reproductive age especially among pregnant women is done to prevent anaemia and improve maternal and child health status. The findings from our study could thus explain that the supplementation did not have any direct impact on improving the iron stores of the children.

In this study, the intermediate factor associated with anaemia was dietary diversity. Dietary diversity helps to measure the overall quality and nutrient adequacy of the diet that may influence blood formation [22]. The current study confirms that children who had poor dietary diversity were 9.15 times more likely to have anaemia as compared to those with good dietary diversity (p<0.001). Our finding is consistent with the results obtained by Woldie and Yigzaw, [9] in an Ethiopian study which revealed that children with poor dietary diversity are 3 times more likely to have anaemia. Our finding also supports results obtained in the study by Kuziga et al. [19] in Ugandan study. In their study, Kuziga et al. [19] reported that children with borderline and poor dietary diversity score had anaemia more than those who had an acceptable dietary diversity score.

Contrary to our findings where significance association was found between dietary diversity and anaemia, another Ghanaian study conducted cross sectionally by Saaka and Galaa [22] found no statistically significant association between the two variables. The differences could probably be due to the different designs adopted by the two studies.

Regarding underlying factors, children whose mothers were aged <20 years or 30-39 years were 4.69 and 2.55 times more likely to develop anaemia, as compared to those who were aged 40 years and above though results were not significant. The findings of this study agree to the results obtained in a study conducted by Borbor et al. [23] in Ghana. Their result also indicated that mothers who were aged 20 years and above were less likely to have their children to be anaemic compared to mothers less than 20 years. Our findings regarding age are also consistent with results obtained in the study by Kweku et al. [12]. In their study, Kweku et al. reported that children of mothers aged 40-49 years were 84% less likely to be anaemic compared to those less than 30 years. The reason for the findings in our study regarding age could probably be that mothers aged 40 years and above are multiparous and thus have experience in child care and feeding practices compared to mothers less than 20 years as found in previous studies [12, 24–27].

We also found that children whose mothers were farmers and traders were less likely to have anaemia compared to those whose mothers were unemployed. This finding is consistent with results of a study by VanBuskirk et al. [28] in Ghana. The authors found that children whose mothers were farmers and traders were less likely to develop anaemia. The possible reason why farmers' children were less likely to have anaemia in our study is that in the Hohoe Municipality, farmers grow iron rich foods such as green leafy vegetables [15]. So, the tendency of feeding their children with enough iron rich foods such as green leafy vegetables is greater as realised in previous studies [20, 29, 30].

## 5. Conclusion

The overall prevalence of anaemia among children aged 6 to 59 months in the Hohoe Municipality was 53.8%. This implies that, out of every 10 children, 5 were anaemic. The biologic, intermediate, and underlying factors that were significantly associated with anaemia comprised maternal iron supplementation, poor dietary diversity, farmers, and traders. Children of mothers aged below 20 years were more likely to be anaemic. Children whose mothers earned income had lower likelihoods of being anaemic compared to those whose mothers were unemployed.

Children whose mothers received iron supplementation were more likely to be anaemic than those who did not. The implication of this finding is that since iron supplementation was provided during pregnancy, its protective capabilities probably waned by six months after delivery and thus could no longer protect the child from developing anaemia. Children with poor dietary diversity were also more likely to be anaemic compared to those with good dietary diversity.

Given that iron supplementation during pregnancy did not protect children against anaemia, we recommend that anaemia intervention programmes in children under five years should target younger and unemployed mothers as well as the intensification of maternal education on good dietary diversity for children.

## 6. Limitation of the Study

The limitation of the study was that data could not be collected to determine seasonal trends. Another limitation was that the study was not able to test for HIV/AIDS, sickle cell disease, and G6PD deficiency and also examine stools for intestinal worm infestations which are known risk factors for anaemia. The study could also not assess the frequency which would have helped to determine the adherence of iron supplementation intake among the mothers and also influence of recall bias which led to the association between maternal iron supplementation and anaemia.

## Figures and Tables

**Table 1:** Sociodemographic characteristics of children aged 6 to 59 months.

Characteristic	Cases [n=70] n (%)	Controls [n=140] n (%)	Total [N=210] N (%)
*Age group (months)*			
6 – 11	5 (7.1)	6 (4.3)	11 (5.2)
12 – 23	10 (14.3)	21 (15.0)	31 (14.8)
24 – 59	55 (78.6)	113 (80.7)	168 (80.0)
*Sex *			
Male	33 (47.1)	66 (47.1)	99 (47.1)
Female	37 (52.9)	74 (52.9)	111 (52.9)
*Rank of Child*			
First	21 (30.0)	47 (33.6)	68 (32.4)
Second	17 (24.3)	32 (22.9)	49 (23.3)
Third	15 (21.4)	25 (17.9)	40 (19.0)
Four and more	17 (24.3)	36 (25.7)	53 (25.2)
*Place of birth *			
Home delivery	17 (24.3)	28 (20.0)	45 (21.4)
Health facility	53 (75.7)	112 (80.0)	165 (78.6)
*Own LLIN*			
No	3 (4.3)	6 (4.3)	9 (4.3)
Yes	67 (95.7)	134 (95.7)	201 (95.7)
*Use LLIN*			
No	14 (20.0)	30 (21.4)	44 (21.0)
Yes	56 (80.0)	110 (78.6)	166 (79.0)
*Blood Film*			
Positive	4 (5.7)	7 (5.0)	11 (5.2)
Negative	66 (94.3)	133 (95.0)	199 (94.8)
*Parasite density*			
Low density	66 (94.3)	134 (95.7)	200 (95.2)
High density	4 (5.7)	6 (4.3)	10 (4.8)
*Fever Status*			
Normal	66 (94.3)	130 (92.9)	196 (93.3)
Fever (≥37.5°C)	4 (5.7)	10 (7.1)	14 (6.7)
*MUAC*			
Normal	65 (92.9)	130 (92.9)	195 (92.9)
Wasted	5 (7.1)	10 (7.1)	15 (7.1)
*Sickle cell *			
No	69 (98.6)	137 (97.9)	206 (98.1)
Yes	1 (1.4)	3 (2.1)	4 (1.9)

**Table 2:** Socio demographic characteristics of mothers.

Characteristics	Cases [N=70] n (%)	Controls [N=140] n (%)	Total [N=210] N (%)
*Maternal Age group (years)*			
40 and above	5 (7.1)	17 (12.1)	22 (10.5)
30 -39	35 (50.0)	49 (35.0)	84 (40.0)
20 – 29	26 (37.2)	70 (50.0)	96 (45.7)
< 20	4 (5.7)	4 (2.9)	8 (3.8)
*Religious Affiliation*			
Christian	68 (97.1)	125 (89.3)	193 (91.9)
Islam	2 (2.9)	15 (10.7)	17 (8.1)
*Tribe *			
Ewe	60 (85.7)	101 (72.1)	161 (76.7)
Guan	9 (12.9)	33 (23.6)	42 (20.0)
Akan and Others	1 (1.4)	6 (4.3)	7 (3.3)
*Educational level *			
None	2 (2.8)	14 (10.0)	16 (7.6)
Primary/JHS	55 (78.6)	106 (75.7)	161 (76.7)
Secondary	13 (18.6)	20 (14.3)	33 (15.7)
*Occupation*			
Unemployed	13 (18.6)	23 (16.4)	36 (17.1)
Civil servant	4 (5.7)	6 (4.3)	10 (4.8)
Farmer	13 (18.6)	39 (27.9)	52 (24.8)
Trader/Hair dresser	40 (57.1)	72 (51.4)	112 (53.3)
*Marital Status*			
Single	15 (21.4)	24 (17.1)	39 (18.6)
Married	55 (78.6)	116 (82.9)	171 (81.4)
*Parity*			
1 -2	30 (42.9)	69 (49.3)	99 (47.1)
3 -4	26 (37.1)	45 (32.1)	71 (33.8)
> 4	14 (20.0)	26 (18.6)	40 (19.0)
*Monthly income*			
<100	45 (64.3)	79 (56.4)	124 (59.0)
100 -200	19 (27.1)	49 (35.0)	68 (32.4)
200+	6 (8.6)	12 (8.6)	18 (8.6)
*Socio economic status *			
Low SES	17 (24.3)	42 (30.0)	59 (28.1)
Middle SES	24 (34.3)	47 (33.6)	71 (33.8)
High SES	29 (41.4)	51 (36.4)	80 (38.1)

**Table 3:** A conditional logistic regression showing association between anaemia and biologic, intermediate, and underlying factors.

	Cases [N=70] n (%)	Controls [N=140] n (%)	COR (95%CI ) p-value	AOR (95%CI ) p-value
*Maternal age (in years)*				
40 and above	5 (7.1)	17 (12.1)	Ref.	Ref.
30 -39	35 (50.0)	49 (35.0)	2.24 (0.78,6.44) 0.132	2.55 (0.66,9.88) 0.175
20 – 29	26 (37.1)	70 (50.0)	1.10 (0.37,3.31) 0.863	0.76 (0.18,3.16) 0.707
< 20	4 (5.7)	4 (2.9)	3.40 (0.63,18.27) 0.155	4.69 (0.46,47.58) 0.191
*Occupation *				
Unemployed	13 (18.6)	23 (16.4)	Ref.	Ref.
Civil servant	4 (5.7)	6 (4.3)	1.18 (0.28,4.97) 0.819	0.60 (0.10,3.74) 0.588
Farmer	13 (18.6)	39 (27.9)	0.65 (0.27,1.53) 0.324	0.17 (0.05,0.60) 0.006*∗*
Trader	26 (37.1)	35 (25.0)	1.05 (0.48,2.32) 0.903	0.21 (0.06,0.74) 0.014
*Child iron supplementation*				
No	58 (82.9)	102 (72.9)	Ref.	Ref.
Yes	12 (17.1)	38 (27.1)	0.52 (0.24,1.13)0.099	0.47 (0.18,1.24) 0.126
*Dietary diversity score*				
Good	33 (47.1)	106 (75.7)	Ref.	Ref.
Poor	37 (52.9)	34 (24.3)	3.89 (1.97,7.68)<0,001*∗*	9.15 (3.13,26.82) <0.001*∗*
*Iron supplementation during pregnancy*				
No	3 (4.3)	16 (11.4)	Ref.	Ref.
Yes	67 (95.7)	124 (88.6)	2.93 (0.82,10.51) 0.100	7.64 (1.41,41.20) 0.018*∗*

*Note.∗* signifies that p-value is significant at 0.05.

## Data Availability

The data used to support the findings of this study are available from the corresponding author upon request.

## References

[1] Semedo R. M. L., Santos M. M. A. S., Baião M. R., Luiz R. R., Da Veiga G. V. (2014). Prevalence of anaemia and associated factors among children below five years of age in cape verde, West Africa. *Journal of Health, Population and Nutrition*.

[2] WHO (2015). *The Global Prevalence of Anaemia 2011*.

[3] McLean E., Cogswell M., Egli I., Wojdyla D., De Benoist B. (2009). Worldwide prevalence of anaemia, WHO Vitamin and Mineral Nutrition Information System, 1993-2005. *Public Health Nutrition*.

[4] Scott S. P., Chen-Edinboro L. P., Caulfield L. E., Murray-Kolb L. E. (2014). The impact of anemia on child mortality: An updated review. *Nutrients*.

[5] Ngesa O., Mwambi H. (2014). Prevalence and risk factors of anaemia among children aged between 6 months and 14 years in Kenya. *Plos One*.

[6] Ofori-Asenso R., Agyeman A. A., Laar A., Boateng D. (2016). Overweight and obesity epidemic in Ghana - A systematic review and meta-analysis. *BMC Public Health*.

[7] GDHS (2014). *Ghana Demographic and Health Survey Report*.

[8] Egbi G., Steiner-Asiedu M., Kwesi F. S. (2014). Anaemia among school children older than five years in the Volta Region of Ghana. *Pan African Medical Journal*.

[9] Simbauranga R. H., Kamugisha E., Hokororo A., Kidenya B. R., Makani J. (2015). Prevalence and factors associated with severe anaemia amongst under-five children hospitalized at Bugando Medical Centre, Mwanza, Tanzania. *BMC Hematology*.

[10] Woldie H., Kebede Y., Tariku A. (2015). Factors associated with anemia among children aged 6-23 months attending growth monitoring at tsitsika health center, wag-himra zone, Northeast Ethiopia. *Journal of Nutrition and Metabolism*.

[11] Ghana Health Service (2016). *Ghana: Landscape Analysis of Anemia and Anemia Programming*.

[12] Kweku M., Parbey P. A., Takramah W., Owusu R., Axame K. W., Tarkang E. (2017). Assessment of the prevalence of anaemia and associated risk factors among children under five years in rural communities ofthe hohoe municipality, Ghana. *International Journal of Nursing Didactics*.

[13] Parbey P. A., Kyei-duodu G., Takramah W. (2017). Prevalence of anaemia and associated risk factors among children under five years in hohoe municipality, Ghana. *Journal of Scientific Research & Reports*.

[14] USAID (2013). *United States Agency International Development: Health Report*.

[15] GSS (2010). *Ghana Statistical Service. Population and Housing Census Report 2010*.

[16] Hohoe Municipal Health Directorate (HMHD) (2018). *Annual Report, 2017*.

[17] Charan J., Biswas T. (2013). Review article how to calculate sample size for different study designs in medical research. *Indian Journal of Psychological Medicine*.

[18] WHO (2010). *Indicators for Assessing Infant and Young Child Feeding Practices Part 2: Measurement*.

[19] Kuziga F., Adoke Y., Wanyenze R. K. (2017). Prevalence and factors associated with anaemia among children aged 6 to 59 months in namutumba district, Uganda: a cross- sectional study. *BMC Pediatrics*.

[20] Gari T., Loha E., Deressa W. (2017). Anaemia among children in a drought affected community in south-central Ethiopia. *Plos One*.

[21] Logan C., Yanina S., Cristina B. C. (2013). Anemia and adherence to oral iron supplementation in a sample of children assisted by the public health network of Rosario, Santa. *Arch ArgentPediatr*.

[22] Saaka M., Galaa S. Z. (2017). How is dietary diversity related to haematological status of preschool children in Ghana?. *Food & Nutrition Research*.

[23] Borbor M., Kumi-kyereme A., Yendaw E., Adu-opong A. (2014). A Study of the determinants of anaemia among under-five children in Ghana. *International Journal of Development Research*.

[24] Leal L. P., Filho M. B., Cabral de LiraI P. C., FigueiroaII J. N., OsórioI M. M. (2011). Prevalence of anaemia and associated factors in children aged 6-59 months in pernambuco, northeastern Brazil. *Revista de SaúdePública*.

[25] Kuziga F., Adoke Y., Wanyenze R. K. (2017). Prevalence and factors associated with anaemia among children aged 6 to 59 months in namutumba district, Uganda: a cross- sectional study. *BMC Pediatrics*.

[26] Matanda D. J., Urke H. B., Mittelmark M. B. (2016). Changes in optimal childcare practices in Kenya: Insights from the 2003, 2008-9 and 2014 demographic and health surveys. *Plos One*.

[27] Legason I. D., Atiku A., Ssenyonga R., Olupot-Olupot P., Barugahare J. B. (2017). Prevalence of anaemia and associated risk factors among children in north-western Uganda: a cross sectional study. *BMC Hematology*.

[28] VanBuskirk K., Ofosu A., Kennedy A., Denno D. (2014). Pediatric anemia in rural ghana: A cross-sectional study of prevalence and risk factors. *Journal of Tropical Pediatrics*.

[29] Baranwal A., Baranwal A., Roy N. (2014). Association of household environment and prevalence of anemia among children under-5 in India. *Frontiers in Public Health*.

[30] Gayawan E., Arogundade E. D., Adebayo S. B. (2014). Possible determinants and spatial patterns of anaemia among young children in nigeria: A bayesian semi-parametric modelling. *International Health*.

